# Association Between Spinal Manipulation, Butalbital Prescription, and Medication Overuse Headache in Adults With Tension‐Type Headache: Retrospective Cohort Study

**DOI:** 10.1002/hsr2.70218

**Published:** 2024-11-29

**Authors:** Robert J. Trager, Timothy J. Williamson, Pratheek S. Makineni, Lindsay H. Morris

**Affiliations:** ^1^ Connor Whole Health University Hospitals Cleveland Medical Center Cleveland Ohio USA; ^2^ Department of Family Medicine and Community Health Case Western Reserve University School of Medicine Cleveland Ohio USA; ^3^ Department of Biostatistics and Bioinformatics Clinical Research Training Program Duke University School of Medicine Durham North Carolina USA; ^4^ UCHealth Spine Center University of Colorado Hospital Aurora Colorado USA; ^5^ Department of Physical Medicine & Rehabilitation, University of Colorado School of Medicine Aurora Colorado USA; ^6^ Case Western Reserve University School of Medicine Cleveland Ohio USA; ^7^ Frances Payne Bolton School of Nursing Case Western Reserve University Cleveland USA

**Keywords:** barbiturates, chiropractic, headache, spinal manipulation

## Abstract

**Background and Aims:**

Butalbital is an acute headache medication commonly prescribed for tension‐type headache (TTH), although discouraged by guidelines due to a risk of medication overuse headache (MOH). Considering spinal manipulative therapy (SMT) may reduce TTH frequency and intensity, we hypothesized adults with TTH receiving chiropractic SMT would be less likely to receive a butalbital prescription over 2 years versus matched controls not receiving SMT. We secondarily compared likelihood of MOH between cohorts.

**Methods:**

We searched a United States medical records database of patients attending academic medical centers for adults with TTH, from 2013 to 2024, excluding those diagnosed with other headaches and seen in inpatient/emergency settings. We divided patients into two cohorts: (1) SMT and (2) non‐SMT, using propensity matching to control for demographics and other variables associated with likelihood of butalbital prescription and MOH.

**Results:**

Three thousand one hundred and sixteen patients remained per cohort after matching. The incidence of butalbital prescription was lower in the SMT cohort compared to the non‐SMT cohort (SMT: 1.7%; non‐SMT: 3.8%), yielding an RR (95% CI) of 0.46 (0.33–0.63; *p* < 0.001). The incidence of MOH was lower in the SMT cohort versus non‐SMT cohort (SMT: 0.5%; non‐SMT: 1.2%), yielding an RR (95% CI) of 0.44 (0.25–0.80; *p* < 0.001).

**Conclusion:**

Adults receiving chiropractic SMT had a significantly lower likelihood of butalbital prescription and, tentatively, MOH compared to matched controls not receiving SMT. These findings support current guideline recommendations favoring SMT in TTH care, though future studies should replicate and compare these findings with other nonpharmacologic clinicians and interventions.

## 1 Background

Tension‐type headache (TTH) is the most common type of headache, with a global prevalence of 26% [1, 2]. TTH is typically characterized by bilateral pressing, tightening head pain, and co‐occurs with neck pain in nearly 90% of patients [3]. It is occasionally treated with butalbital or butalbital‐containing medications for acute/abortive purposes [4, 5, 6]. However, this has been discouraged by practice guidelines due to poor efficacy and potential complications such as withdrawal, overuse, and medication overuse headache (MOH) [2, 7, 8, 9]. Patients also seek spinal manipulative therapy (SMT) for TTH relief [10, 11, 12]. While evidence supports the efficacy of SMT for TTH [13, 14], its association with butalbital prescription and MOH remains unknown [15].

First‐line medications for TTH include acetaminophen and aspirin [3, 7, 8, 16]. Butalbital, a barbiturate and sedative/hypnotic medication, is a second‐line therapy but carries the risk of cognitive impairment, dependence/addiction, and headache chronification [7, 9]. In addition, patients may develop MOH, described as a worsening of the primary headache after overuse or discontinuation of acute headache medication, and occurring at least 15 days per month [17]. Common butalbital‐containing medications used for headaches often include caffeine and either acetaminophen or aspirin. Butalbital‐containing medications have not demonstrated efficacy for headaches in comparison to other treatments or placebo [18].

Several studies suggest that butalbital is commonly prescribed for TTH. An examination of a US data set of ambulatory visits for nonserious, non‐migraine headaches found that either opioids or barbiturates were prescribed in approximately 15% of cases [4]. According to data from a single US academic headache center, one in five patients was currently using either opioids or barbiturates, while more than half of patients had been prescribed an opioid or barbiturate [5]. Finally, another study highlighted that butalbital prescription was more common than opioid prescription for TTH [6].

Chiropractors are non‐pharmacologic clinicians who frequently use SMT to manage musculoskeletal disorders [11]. Systematic reviews have found evidence that SMT may reduce TTH intensity and frequency compared to sham interventions or no treatment [13, 14]. In addition, SMT is recommended for TTH by the US Centers for Disease Control [19]. Considering SMT may benefit TTH, it remains plausible that patients receiving SMT may be less inclined to seek medications for acute TTH relief such as butalbital. As butalbital is not a first‐line medication for TTH, examination of its prescription could reflect acute exacerbations of recalcitrant headaches and/or medication guideline non‐adherence.

This study addresses gaps in the TTH literature by examining the association between SMT and butalbital prescription and MOH. We hypothesized that adults receiving chiropractic SMT for TTH would have a reduced likelihood of receiving a butalbital prescription over a 1‐year follow‐up compared to matched controls not receiving SMT, and secondarily compared the likelihood of MOH between cohorts.

## 2 Materials and Methods

### 2.1 Study Design

This study implemented a retrospective cohort design with a data range spanning 11 years to present coinciding with US headache recommendations discouraging butalbital as a first‐line intervention [9]. We adhered to a registered protocol [20]. Inclusion of patients ended 2 years before the query date (April 15, 2024), allowing for ascertainment of the outcomes. Study reporting conforms to the Strengthening the Reporting of Observational Studies in Epidemiology (STROBE) guideline [21]. This study used deidentified, anonymized data from TriNetX (TriNetX Inc. Cambridge, MA, US) obtained via the University Hospitals Clinical Research Center Honest Broker. The University Hospitals Institutional Review Board (IRB; Cleveland, OH, US) considered the present study “not human subjects research,” therefore not requiring review board approval or patient consent (IRB number: STUDY20241256). In addition, TriNetX has received an exemption from Western IRB, which waives the need for patient consent.

The US TriNetX network includes over 124 million individuals from 89 academic medical centers and their affiliated community and ambulatory offices [22, 23], and approximately 145,000 unique patients receiving chiropractic SMT at the time of our query. Data are routinely collected and can be examined to conduct longitudinal and retrospective research [22, 23]. This data resource may be searched using standardized terminology such as the International Classification of Diseases, 10th Edition (ICD‐10) diagnosis codes, which are converted automatically to 9th Edition whenever necessary [22, 23]. Data span demographics, diagnoses, procedures, laboratory values, and medications [23]. TriNetX monitors standard levels of data quality with respect to conformance, consistency, and completeness [22, 23] and the data have demonstrable medication completeness meeting at least 87% [24]. Data are deidentified via contractual and technical safeguards [22, 23].

TriNetX complies with the Health Insurance Portability and Accountability Act (HIPAA), the US federal law that protects healthcare data privacy and security. TriNetX is certified to the International Organization for Standardization 27001:2013 standard and maintains an Information Security Management System to ensure the protection of the healthcare data it has access to and to meet the requirements of the HIPAA Security Rule. Data are deidentified per the de‐identification standard defined in Section §164.514(a) of the HIPAA Privacy Rule. The process of de‐identifying data is attested to through a formal determination by a qualified expert as defined in Section §164.514(b)(1) of the HIPAA Privacy Rule. The TriNetX network contains data provided by participating healthcare organizations, each of which represents and warrants that it has all necessary rights, consents, approvals, and authority to provide the data to TriNetX under a Business Associate Agreement, so long as their name remains anonymous as and their data are utilized for research purposes. The data are attenuated to ensure that they do not include sufficient information to identify the healthcare organization that contributed specific patient information.

We used the natural language processing software available within TriNetX (Averbis, Freiburg im Breisgau, DE) [23], which uses algorithms to extract information from unstructured clinical data such as chart notes and test results [25, 26]. This software has demonstrated adequate reliability and accuracy compared to manual chart review, with a Kappa value of 0.79 (good) [26, 27]. Our use of natural language processing aimed to enhance the application of selection criteria and identification of propensity‐matched variables.

### 2.2 Participants

We included adults aged at least 18 years with a diagnosis of TTH (ICD‐10: G44.2). To standardize and improve data completeness and healthcare utilization between cohorts, we required a healthcare visit within 1 month and 2 years preceding, and within 1 day and 1 year following the index date of inclusion.

We divided patients into two cohorts beginning at an index date defined as (1) SMT; the first co‐occurrence of any of the Current Procedural Terminology (CPT) codes for chiropractic SMT (98940, 98941, 98942) and TTH diagnosis code, and (2) non‐SMT; the first co‐occurrence of an ambulatory office evaluation (CPT: 1013626) with a TTH diagnosis code.

We excluded patients at risk for serious secondary or other primary headache types [28]: those with brain tumors, cerebral infarction, complicated headache syndromes, MOH, migraine, posttraumatic headache or intracranial injury, temporal arteritis, transient ischemic attack, trigeminal autonomic cephalgia, trigeminal neuralgia, and vascular headache. To more thoroughly exclude migraineurs, we also excluded those prescribed antimigraine medications. To help exclude individuals with serious secondary headache etiologies, we excluded patients in emergency, inpatient, or critical care settings on the index date. Exclusions are summarized in Supporting Information S1: Table 1.

### 2.3 Variables

We used propensity matching to reduce bias [29], balancing key variables present within 1 year preceding the index date (Supporting Information S1: Table 2). We matched medications that are often used for TTH which may influence MOH likelihood: acetaminophen, antidepressants, aspirin, butalbital, caffeine, ibuprofen, and skeletal muscle relaxants [8, 17, 30]. We matched additional variables associated with MOH including age, sex, chronic pain, anxiety, depression, eating disorders, obesity, gastrointestinal disorders, neck pain, sedatives, sleep disorders, substance use disorders, a measure of adverse socioeconomic and psychosocial circumstances, and tobacco use [17, 31, 32, 33]. We matched pregnancy, which would reduce the likelihood of butalbital prescription [34]. We also matched race and ethnicity, which may generally influence prescribing behaviors for headaches [35]. Finally, we matched any prescription medication, to control for potential differences in pharmacological care preferences between cohorts [36].

For our primary outcome, we identified butalbital prescriptions (RxNorm: 19860), rather than the broader category of barbiturates. Barbiturates may be used to treat other conditions such as seizures or for anesthesia, whereas butalbital is primarily used for headaches [34].

We used an outcome assessment window of 2 years to give greater insights into the long‐term management of TTH and the slow development of MOH (ICD‐10: G44.4) [31]. The outcome assessment window began the day following the index date, to further exclude patients already diagnosed with MOH, and because our focus is on long‐term, longitudinal outcomes rather than immediate care.

### 2.4 Required Study Size

We calculated a total required sample size of 5076 using data from a prior study regarding MOH [17]. Considering this outcome is less common than butalbital prescription, it allowed us to adequately power the study for both our primary and secondary outcomes. We used G*Power (Kiel University, DE), and Z‐tests to determine a difference between two independent proportions (0.015 vs. 0.030), two‐tailed α error of 0.05, a power of 0.95, and an allocation ratio of 1.

### 2.5 Statistical Methods

We used built‐in features of the TriNetX software for statistical analysis. Propensity scores were derived using logistic regression to estimate the log odds of non‐SMT cohort assignment, ranging from 0 (lowest likelihood) to 1 (highest likelihood). Matching will implement a greedy nearest‐neighbor algorithm, a 1:1 matching ratio, using a calliper of 0.01 pooled standard deviations. Baseline characteristics will be compared using Pearson χ^2^ and independent‐sample *t*‐tests. Standardized mean difference (SMD) was used to assess between‐cohort balance, with a threshold of < 0.1 [37]. We did not make imputations for missing data. We used R (version 4.2.2, Vienna, AT) [38]. To calculate 95% confidence intervals (CIs) and the ggplot2 package [39] to plot propensity score density and cumulative incidence of butalbital prescription as a sensitivity analysis. The risk ratios (RR) for butalbital prescription were calculated by dividing the incidence proportion in the SMT cohort by the non‐SMT cohort. As a secondary outcome, we calculated the RR for MOH. We calculated p‐values for RRs using the chi‐square test and evaluated significance using a two‐tailed alpha level of *p* ≤ 0.05.

For additional secondary outcomes, we assessed the adequacy of propensity matching by calculating RRs for negative control outcomes that we expected to remain uninfluenced by SMT [40], including antibiotics and proton pump inhibitors. We aimed for point estimates for each outcome suggestive of between cohort balance (i.e., 0.73 ≥ RR ≤ 1.38) [41]. We also examined the median, mean, and standard deviation of follow‐up SMT visits.

## 3 Results

### 3.1 Participants

Before propensity matching, there were 3118 patients in the SMT cohort and 141,039 in the non‐SMT cohort. After matching, 3116 patients remained per cohort. Before matching, patients in the SMT cohort were more often female and White, less often Hispanic or Latino, Black or African American or other race and a greater proportion of patients were diagnosed with cervicalgia and chronic pain (SMD > 0.1; Table 1). Prior prescriptions also varied, with the SMT cohort having a greater proportion of those prescribed aspirin or antidepressants, yet a lower proportion of those prescribed butalbital or caffeine (SMD > 0.1). After matching, all key variables were ideally matched (SMD < 0.1). A greater proportion of patients in the SMT cohort had received chiropractic care for a non‐TTH disorder before the index date of inclusion, yet previous chiropractic care was not matched and only reported for descriptive purposes.

**Table 1:** Baseline characteristics.

	Before matching			After matching		
Variable (n (%) or mean (SD))	SMT	Non‐SMT	SMD	SMT	Non‐SMT	SMD
N	3118	141,039	NA	3116	3116	NA
Demographics						
Age at Index	47.8 (16.9)	47.6 (16.2)	0.013	47.9 (16.9)	47.8 (16.4)	0.004
Female	2375 (76%)	101207 (72%)	0.101	2373 (76%)	2385 (77%)	0.009
Male	743 (24%)	33568 (24%)	0.001	743 (24%)	729 (23%)	0.011
Hispanic or Latino	65 (2%)	18609 (13%)	0.428	65 (2%)	67 (2%)	0.004
Not Hispanic or Latino	2708 (87%)	93309 (66%)	0.503	2706 (87%)	2712 (87%)	0.006
American Indian or Alaska Native	16 (1%)	406 (< 1%)	0.036	16 (1%)	12 (< 1%)	0.019
Asian	26 (1%)	5795 (4%)	0.212	26 (1%)	27 (1%)	0.003
Black or African American	125 (4%)	25736 (18%)	0.465	125 (4%)	119 (4%)	0.010
Native Hawaiian or Other Pacific Islander	10 (< 1%)	882 (1%)	0.044	10 (< 1%)	10 (< 1%)	0.000
White	2548 (82%)	83227 (59%)	0.513	2546 (82%)	2525 (81%)	0.017
Other Race	37 (1%)	6272 (4%)	0.198	37 (1%)	48 (2%)	0.030
Diagnoses						
Adverse socioeconomic circumstances	62 (2%)	6997 (5%)	0.163	62 (2%)	65 (2%)	0.007
Anxiety‐related disorders	982 (31%)	43225 (31%)	0.018	981 (31%)	988 (32%)	0.005
Cervicalgia	1580 (51%)	21068 (15%)	0.823	1578 (51%)	1609 (52%)	0.020
Chronic pain, not elsewhere classified	615 (20%)	21101 (15%)	0.126	615 (20%)	629 (20%)	0.011
Diseases of the digestive system	1237 (40%)	57994 (41%)	0.029	1237 (40%)	1231 (40%)	0.004
Mood disorders	750 (24%)	34154 (24%)	0.004	749 (24%)	724 (23%)	0.019
Nicotine dependence	181 (6%)	13978 (10%)	0.153	181 (6%)	179 (6%)	0.003
Overweight and obesity	526 (17%)	25437 (18%)	0.031	526 (17%)	501 (16%)	0.022
Psychoactive substance use	265 (8%)	19634 (14%)	0.172	265 (9%)	261 (8%)	0.005
Pregnancy, childbirth, and the puerperium	215 (7%)	8765 (6%)	0.028	214 (7%)	216 (7%)	0.003
Sleep disorders	604 (19%)	24306 (17%)	0.055	604 (19%)	588 (19%)	0.013
Tobacco use	37 (1%)	7922 (6%)	0.246	37 (1%)	41 (1%)	0.012
Prior treatments						
Medication(s) (any)	2881 (92%)	129229 (92%)	0.028	2879 (92%)	2846 (91%)	0.039
Acetaminophen	992 (32%)	49214 (35%)	0.065	991 (32%)	937 (30%)	0.037
Antidepressants	1218 (39%)	44168 (31%)	0.163	1216 (39%)	1166 (37%)	0.033
Aspirin	455 (15%)	15822 (11%)	0.101	453 (15%)	435 (14%)	0.017
Benzodiazepines	638 (20%)	32124 (23%)	0.056	638 (20%)	652 (21%)	0.011
Butalbital	45 (1%)	11090 (8%)	0.308	45 (1%)	58 (2%)	0.033
Caffeine	100 (3%)	14042 (10%)	0.275	100 (3%)	115 (4%)	0.026
Ibuprofen	672 (22%)	27463 (19%)	0.052	670 (22%)	648 (21%)	0.017
Opioid analgesics	902 (29%)	48602 (34%)	0.119	902 (29%)	871 (28%)	0.022
Sedatives/hypnotics	230 (7%)	9026 (6%)	0.039	230 (7%)	233 (7%)	0.004
Skeletal muscle relaxants	698 (22%)	32402 (23%)	0.014	698 (22%)	672 (22%)	0.020
Chiropractic care* (spinal or extraspinal manipulation)	2668 (86%)	93 (0%)	3.432	2666 (86%)	57 (2%)	3.148

*Note:* Reported for descriptive purposes only and not matched (*). Counts of 10 should be interpreted with caution as TriNetX rounds count 1–9 up to 10 for de‐identification purposes.

Abbreviation: SMD, Standardized mean difference.

### 3.2 Descriptive Data

**Figure 1:**
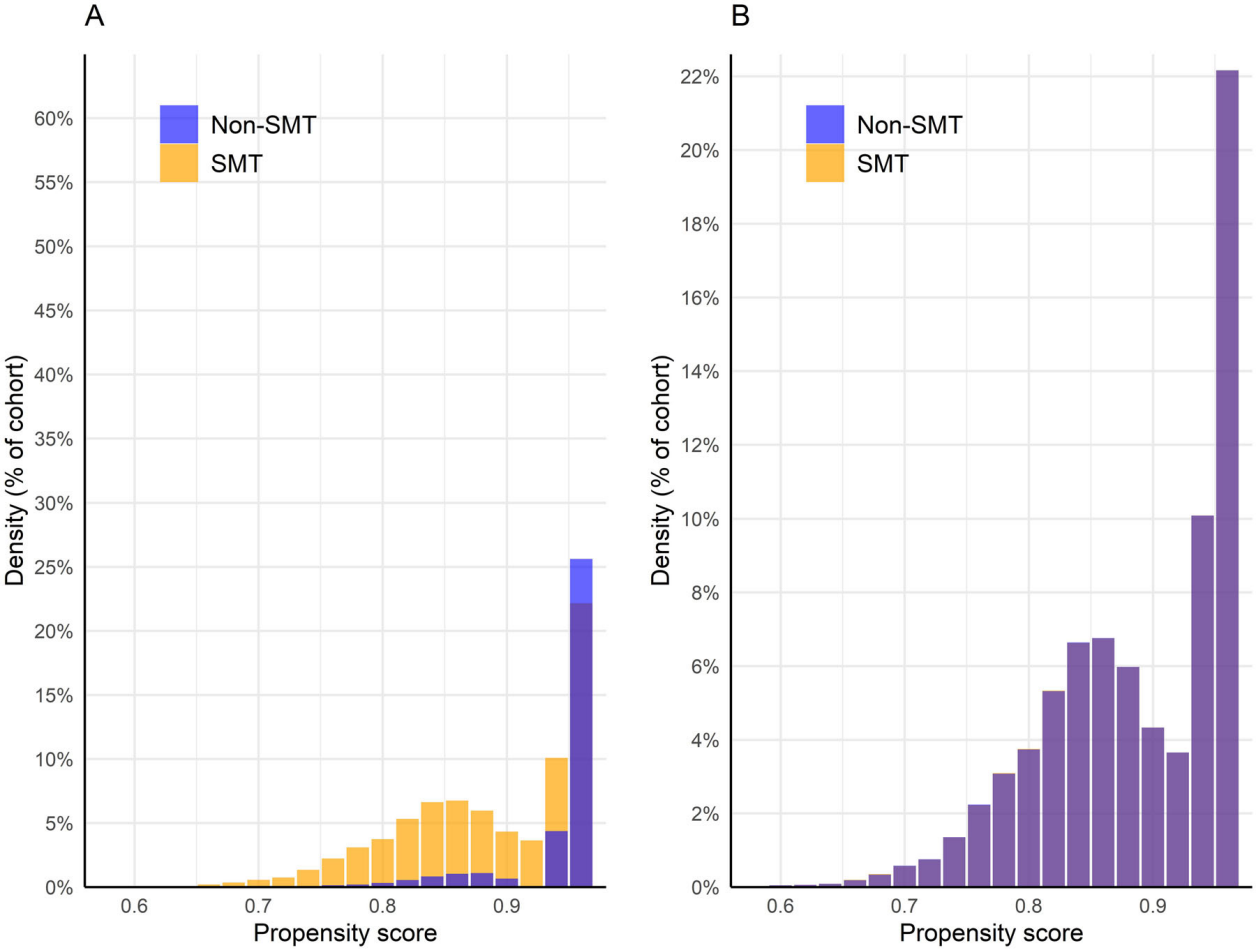
Propensity score density graph. Propensity scores before (A) and after (B) matching. The orange bars represent the spinal manipulative therapy (SMT) cohort while the blue bars represent the non‐SMT cohort. After matching, propensity score densities overlap closely, suggesting adequate covariate balance.

There was an adequate mean number of data points per patient per cohort (SMT: 4180; non‐SMT: 3629). After matching, the proportion of unknown demographic variables was similar between cohorts: unknown ethnicity (both cohorts: 11%; SMD = 0.008), unknown sex (SMT: 0%; non‐SMT: < 1%; SMD = 0.80), and unknown age (both cohorts: 0%, SMD = 0). Plotted post‐matching propensity score densities overlapped, suggesting that covariates were adequately balanced (Figure 1). Together, these findings suggest that there were minimal between‐cohort differences with respect to data density, completeness, and covariate balance.

### 3.3 Primary Outcome

**Figure 2:**
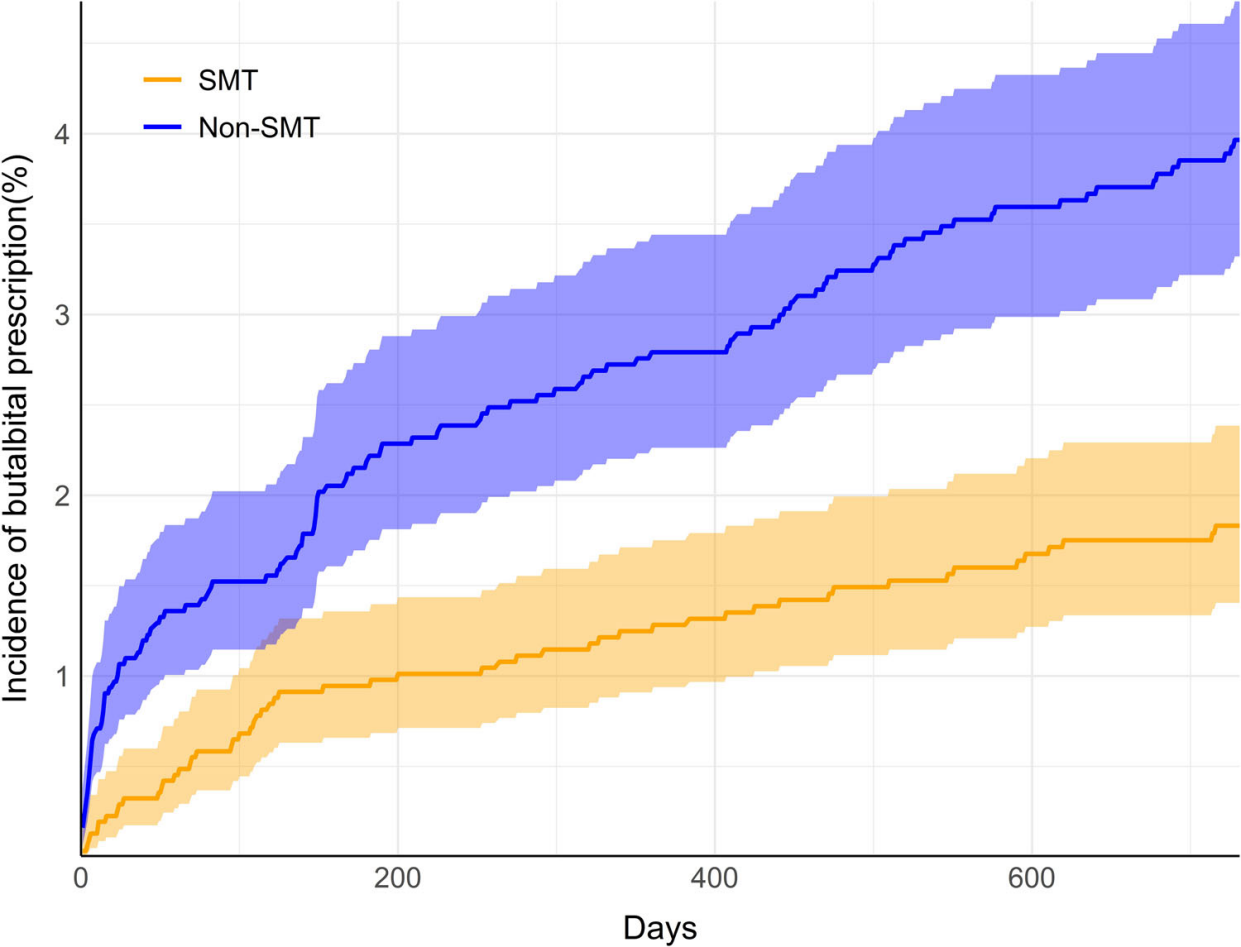
Cumulative incidence of butalbital prescription. Incidence curves in the spinal manipulative therapy cohort (SMT; orange) and non‐SMT (blue) are shown through two years' follow‐up (730 days). Shaded regions highlight 95% confidence intervals.

The incidence of butalbital prescription through 2 years' follow‐up from the index date of inclusion was lower in the SMT cohort compared to the non‐SMT cohort (Table 2). After propensity matching, 1.7% of the SMT cohort had received a butalbital prescription, compared to 3.8% of the non‐SMT cohort, translating to an RR (95% CI) of 0.46 (0.33–0.63; *p* < 0.001). A plot highlighted a curvilinear increase in cumulative incidence of butalbital prescription in the non‐SMT cohort compared to a more linear pattern in the non‐SMT cohort (Figure 2).

**Table 2:** Primary outcome of butalbital prescription.

	Before matching		After matching	
	SMT	Non‐SMT	SMT	Non‐SMT
Number of patients	3118	141,039	3116	3116
Butalbital, n	54	8,232	54	118
Butalbital, % (95% CI)	1.7 (1.3–2.2)	5.8 (5.7–6.0)	1.7 (1.3–2.2)	3.8 (3.1–4.5)
RR (95% CI)	0.30 (0.23–0.39; *p* < 0.001)	(Reference)	0.46 (0.33–0.63; *p* < 0.001)	(Reference)

Abbreviations: 95% CI, 95% confidence intervals; RR, risk ratio; SMT, spinal manipulative therapy.

### 3.4 Secondary Outcomes

**Figure 3:**
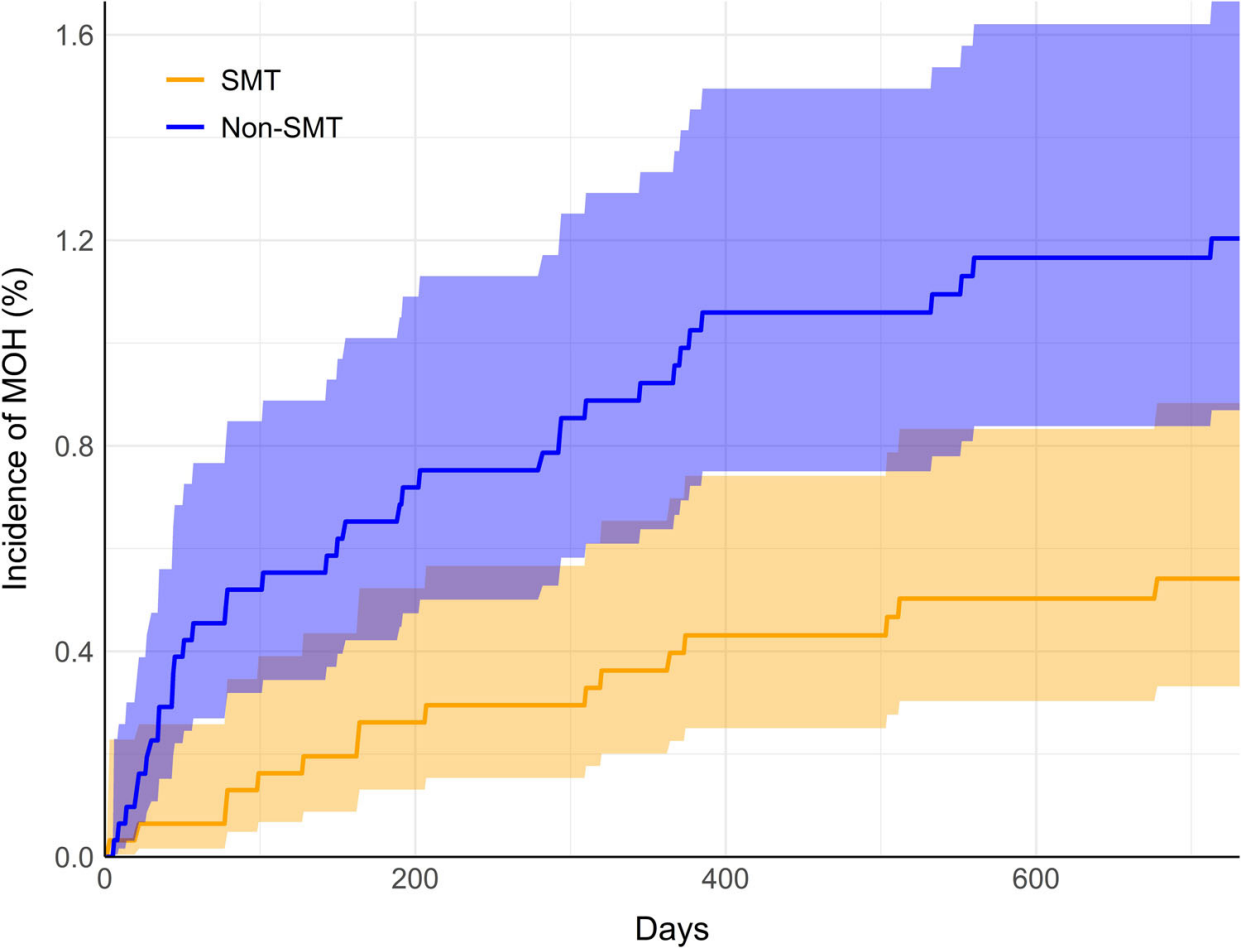
Cumulative incidence of medication overuse headache (MOH). Incidence curves in the spinal manipulative therapy cohort (SMT; orange) and non‐SMT (blue) are shown through two years' follow‐up (730 days). Shaded regions highlight 95% confidence intervals.

The incidence of MOH through 2 years' follow‐up from the index date of inclusion was lower in the SMT cohort compared to the non‐SMT cohort (Table 3). After propensity matching, 0.5% of the SMT cohort had been diagnosed with MOH, compared to 1.2% of the non‐SMT cohort, translating to an RR (95% CI) of 0.44 (0.25–0.80; *p* < 0.001). A plot highlighted an earlier increase in the cumulative incidence of MOH compared to the non‐SMT cohort (Figure 3).

**Table 3:** Secondary outcome of medication overuse headache.

	Before matching		After matching	
	SMT	Non‐SMT	SMT	Non‐SMT
Number of patients	3118	141,039	3116	3116
MOH, n	16	1068	16	36
MOH, % (95% CI)	0.5 (0.3–0.8)	0.8 (0.7–0.8)	0.5 (0.3–0.8)	1.2 (0.8–1.5)
RR (95% CI)	0.68 (0.41–1.11; *p* < 0.1186)	(Reference)	0.44 (0.25‐0.80; *p* < 0.001)	(Reference)

Abbreviations: 95% CI, 95% confidence intervals; MOH, medication overuse headache; RR, risk ratio; SMT, spinal manipulative therapy.

Follow‐up SMT visits were common in the SMT cohort, with 73% of patients having more than one follow‐up visit, at a mean of 15 visits [SD = 17] and median of 9.

Prescription of negative outcome control medications was similar when comparing the SMT to the non‐SMT cohort, with antibiotics prescribed in 25.1% versus 23.6% (RR = 1.07; 95% CI: 0.98,1.16; *p* = 0.1481) and proton pump inhibitors prescribed in 29.9% versus 28.8% (RR = 1.04; 95% CI: 0.96,1.12; *p* = 0.3302). These findings provide further evidence that cohorts were adequately balanced through propensity matching.

## 4 Discussion

The present study findings support our hypothesis that adults receiving initial SMT for TTH have a significantly reduced likelihood of butalbital prescription compared to matched controls not receiving SMT through 2 years' follow‐up, as well as a reduced likelihood of MOH. Analysis of cumulative incidence suggests that between‐cohort differences in outcomes of butalbital prescription and MOH may stem from the initial care‐receiving period and persist thereafter. These findings suggest there is potential usefulness of SMT in managing TTH extending beyond improvement in headache frequency or intensity, and reinforce guidelines recommending SMT for TTH patients [19, 42].

The present findings could be explained as a function of care pathways. Chiropractors are nonpharmacologic providers and do not prescribe butalbital, therefore they are not faced with pressure to prescribe medications for TTH. A previous scoping review found evidence to suggest that prioritizing a nonmedical provider at the first point of care for low back pain was associated with a reduction in opioid prescriptions [43]. It is plausible but unclear whether this mechanism would also apply to butalbital prescription and MOH in the context of TTH. Regardless, the present study findings are likely not explained by chiropractic patients' desire to avoid prescription medications. As we propensity matched on past medications, over 90% of patients in both cohorts were already prescribed medications at baseline.

The observed reduction in butalbital prescriptions may be explained by the clinical benefits of SMT for TTH. Evidence from recent systematic reviews suggests that SMT may reduce the pain intensity of TTH [14, 44, 45, 46]. Accordingly, this may reduce patients' likelihood of subsequent specialist visits or encounters where they may be prescribed butalbital. Secondarily, considering butalbital is a risk factor for development of MOH [17], any reduction in butalbital prescription could correspond with a reduction in MOH likelihood.

The present findings could have important implications. While both outcomes reflected a statistically significant decrease in RR with SMT, the absolute risk differences were relatively small. Despite being a smaller magnitude between cohorts, differences in the likelihood of MOH (0.7%) may be more clinically relevant than differences in butalbital prescription (2.1%). MOH is a chronic, debilitating headache that adversely affects quality of life, work, and income, and is more impactful than TTH itself [17]. MOH is difficult to treat and requires a multidisciplinary approach [17]. Accordingly, a small but significant decrease in the likelihood of MOH may be clinically relevant.

Future studies could build on the present findings by examining the likelihood of butalbital prescription across a range of clinician types, including chiropractors and other nonpharmacologic practitioners (e.g., acupuncturists, physical therapists) as well as prescribing clinicians (e.g., neurologists, primary care physicians). This would help determine if our observed association is explained by a general effect of nonpharmacologic care or is more related to the SMT intervention. Second, this study could be repeated while also considering the costs of care attributed to MOH. Considering the low incidence of MOH, a prospective randomized controlled trial may be challenging unless multicenter recruitment is possible.

### 4.1 Strengths and Limitations

The present study was strengthened by adherence to a registered protocol [20], by having a multidisciplinary team, use of a large national data set, and propensity matching strategy. Furthermore, there are several markers of validity of our study, such as the demographics including a majority of females, among whom TTH is most prevalent, a moderately high comorbid neck pain prevalence, and past use of several medications used to treat TTH among both cohorts [3]. While specific estimates within TTH populations are limited, our incidence of butalbital prescription and MOH appear in agreement with the reported literature [4, 5, 6, 31].

However, several limitations should be noted. As an observational study, we cannot conclude that SMT was causative of reductions in butalbital prescription or MOH. There may have been unmeasured confounding related to the severity of the TTH, headache days per month, socioeconomic markers, clinician type initially seen (i.e., neurologist, primary care, pediatrician, pain management), over‐the‐counter medications not documented in the medical record, drug interactions, off‐label prescription of butalbital, and lifestyle factors, including stress, physical activity levels, and sleep quality [5, 17, 31, 32]. In general, patient characteristics that may influence health opportunities and outcomes such as occupation, education, and religion are poorly represented in the data set. SMT is a broad term referring to the use of manual therapies directed to the joints of the spine [47, 48, 49, 50]. Considering our study focused solely on chiropractic SMT, our findings may not be generalizable to all SMT techniques, which may differ in real‐world administration, effectiveness, safety, and utilization for TTH from SMT used by chiropractors in the US [47, 48]. Patients' clinical chart information could be incorrect, leading to documentation bias with selection criteria and propensity matching. Examination of the likelihood of specialty‐specific encounters over follow‐up was not possible given the constraints of the data set.

We were unable to validate the query given our use of deidentified multicenter data. Study findings may only generalize to academic settings in the United States, considering the use of SMT and/or butalbital may vary in other countries.

## 5 Conclusion

Our findings reveal a significant reduction in likelihood of both butalbital prescription and, tentatively, MOH, through 2‐years' follow‐up among adults with TTH receiving SMT compared to matched controls. These findings reinforce clinical practice guidelines already recommending SMT for TTH. However, additional research is needed to corroborate our results and examine the association between a broader variety of nonpharmacologic interventions and butalbital prescription and MOH.

## Author Contributions

**Robert J. Trager:** conceptualization, methodology, software, formal analysis, investigation, resources, data curation, writing–original draft, writing–review and editing, visualization, supervision, project administration, funding acquisition. **Timothy J. Williamson:** conceptualization, methodology, investigation, writing–review and editing. **Pratheek S. Makineni:** conceptualization, methodology, investigation, writing–review and editing. **Lindsay H. Morris:** conceptualization, methodology, investigation, writing–review and editing.

## Disclosure

The lead author Robert J. Trager affirms that this manuscript is an honest, accurate, and transparent account of the study being reported; that no important aspects of the study have been omitted; and that any discrepancies from the study as planned (and, if relevant, registered) have been explained.

## Ethics Statement

This study used de‐identified, deidentified, anonymized data from TriNetX (TriNetX Inc., Cambridge, MA, US) obtained via the University Hospitals Clinical Research Center Honest Broker. The University Hospitals Institutional Review Board (Cleveland, OH, US) considered the present study “not human subjects research,” therefore not requiring review board approval or patient consent (IRB number: STUDY20241256).

## Conflicts of Interest

The authors declare no conflicts of interest.

## Supporting information

Supporting information.

## Data Availability

The minimal, de‐identified, deidentified, aggregated data for baseline characteristics and plots of propensity score density and cumulative incidence of butalbital prescription and MOH are available in Figshare (https://doi.org/10.6084/m9.figshare.25757271) [51].
