# Supplementary material for: Association Between Spinal Manipulation, Butalbital Prescription, and Medication Overuse Headache in Adults With Tension‐Type Headache: Retrospective Cohort Study
Source: Health Sci Rep. 2024 Nov 29;7(12):e70218. doi: 10.1002/hsr2.70218 (PMC11605481; doi:10.1002/hsr2.70218)
Supplement: Supplementary file 1 — Supporting information. [file HSR2-7-e70218-s001.docx]

# Supporting Information

*Table 1: Exclusions for both cohorts*

| **Variable** | **Definition** | **Time window (days)** |
| --- | --- | --- |
| Diagnoses (ICD-10) | | |
| C69-C72 | Malignant neoplasms of eye, brain, and other parts of central nervous system | (-730, 0) |
| D33 | Benign neoplasm of brain and other parts of central nervous system | (-730, 0) |
| G43 | Migraine | (-730, 0) |
| G44.0 | Cluster headaches and other trigeminal autonomic cephalgias | (-730, 0) |
| G44.1 | Vascular headache, not elsewhere classified | (-730, 0) |
| G44.3 | Post-traumatic headaches | (-730, 0) |
| G44.4 | Drug-induced headache, not elsewhere classified (medication overuse headache) | (-730, 0) |
| G44.5 | Complicated headache syndromes (includes new daily persistent headache) | (-730, 0) |
| G45 | Transient cerebral ischemic attacks and related syndromes | (-730, 0) |
| G46 | Vascular syndromes of brain in cerebrovascular diseases | (-730, 0) |
| G50 | Disorders of trigeminal nerve | (-730, 0) |
| I60-I69 | Cerebrovascular diseases | (-730, 0) |
| M31.6 | Other giant cell arteritis | (-730, 0) |
| M31.5 | Giant cell arteritis with polymyalgia rheumatica | (-730, 0) |
| S06 | Intracranial injury | (-730, 0) |
| Z86.73 | Personal history of transient ischemic attack, and cerebral infarction without residual deficits | (-730, 0) |
| Medications | |  |
| N02C (ATC) | Antimigraine preparations | (-730, 0) |
| Visits |  |  |
| 1013729 (CPT) | Critical care services |  |
| NA | Visit: Emergency | (0, 0) |
| NA | Visit: Inpatient encounter | (0, 0) |
| Abbreviations: Anatomical Therapeutic Chemical Classification (ATC); Current Procedural Terminology (CPT); International Classification of Diseases, 10^th^ Edition (ICD-10); not applicable (NA; TriNetX uses a custom process to identify these visits and they are labeled according to the definition). | | |

Table 2: Variables controlled for in propensity score matching

| **Variable/Code** | **Description** |
| --- | --- |
| Demographics | Patient age, sex, race, and ethnicity |
| Diagnoses (ICD-10) | |
| E66 | Overweight and obesity |
| F10-F19 | Mental and behavioral disorders due to psychoactive substance use |
| F30-F39 | Mood disorders (includes depression) |
| F40-F48 | Anxiety, dissociative, stress-related, somatoform and other nonpsychotic mental disorders |
| F50 | Eating disorders |
| G47 | Sleep disorders |
| G89.2 | Chronic pain, not elsewhere classified |
| K00-K95 | Diseases of the digestive system |
| M54.2 | Cervicalgia |
| O00-O9A | Pregnancy, childbirth, and the puerperium |
| Z72.0 | Tobacco use |
| Prescription medications | |
| 161 (RxNorm) | Acetaminophen |
| 1191 (RxNorm) | Aspirin |
| 1886 (RxNorm) | Caffeine |
| 5640 (RxNorm) | Ibuprofen |
| 19860 (RxNorm) | Butalbital |
| CN101 (VA) | Opioid analgesics |
| CN203 (VA) | Benzodiazepine derivative sedative/hypnotics |
| CN309 (VA) | Sedatives/hypnotics, other (includes nonbenzodiazepines) |
| CN600 (VA) | Antidepressants |
| MS200 (VA) | Skeletal muscle relaxants |
| VA000 (VA) | Medications (any) |
| Social determinants of health (ICD-10) | |
| Z55-65 | Adverse socioeconomic and psychosocial circumstances |
| Abbreviations: Normalized names for clinical drugs (RxNorm); International Classification of Diseases, 10^th^ Edition (ICD-10); Veterans Health Administration National Drug File (VA) | |
